# Epigenetic and Transcriptional Signaling in Ewing Sarcoma—Disease Etiology and Therapeutic Opportunities

**DOI:** 10.3390/biomedicines10061325

**Published:** 2022-06-05

**Authors:** Mingli Li, Chun-Wei Chen

**Affiliations:** 1 Department of Systems Biology, Beckman Research Institute, City of Hope, Duarte, CA 91010, USA; mingli@coh.org; 2 City of Hope Comprehensive Cancer Center, Duarte, CA 91010, USA

**Keywords:** Ewing sarcoma, fusion protein, EWSR1-FLI1, EWS-FLI1, epigenetic regulation, transcriptional regulation, functional genomic screens, high throughput screens

## Abstract

Ewing sarcoma (EwS), a type of bone and soft tissue tumor, is mainly driven by the expression of the fusion protein EWSR1-FLI1. Upon binding to chromatin, EWSR1-FLI1 reprograms the epigenetic state, alters gene expression, and thus leads to tumorigenesis. Considerable studies have investigated the epigenomic and transcriptomic profiling of EwS. Nevertheless, a comprehensive view of therapeutic targets is still lacking. This review discusses the epigenetic and transcriptional alterations reported in EwS. Specifically, we discuss the binding characteristics of EWSR1-FLI1 on chromatin, the mechanisms of EWSR1-FLI1 in reprograming epigenome, and EWSR1-FLI1-induced transcriptional alterations. Moreover, we summarize the chemical, RNAi, and CRISPR-cas9 high throughput screens conducted in EwS with the goal of assisting in the development of novel therapies to treat this aggressive disease.

## 1. Introduction

In the last decade, next generation sequencing (NGS) technologies have improved our understanding of cancer mechanisms at a higher molecular level [1]. For example, we can now identify somatic genetic changes at the genome level. Moreover, comparison of the variable expression of genes within and across individuals is now possible. We can investigate transcriptional changes at the whole transcriptome level and identify binding of chromatin modifiers on chromatins at the whole epigenome level.

Ewing sarcoma (EwS) is the second most common primary malignant bone tumor in pediatric patients [2]. It accounts for around 10% to 15% of all primary bone tumors in children and adolescents. In localized cases, the survival rates are around 70% after five years and 30% after ten years. However, when metastasis is diagnosed, the five-year survival rates drop sharply to 25%.

**Figure 1:**
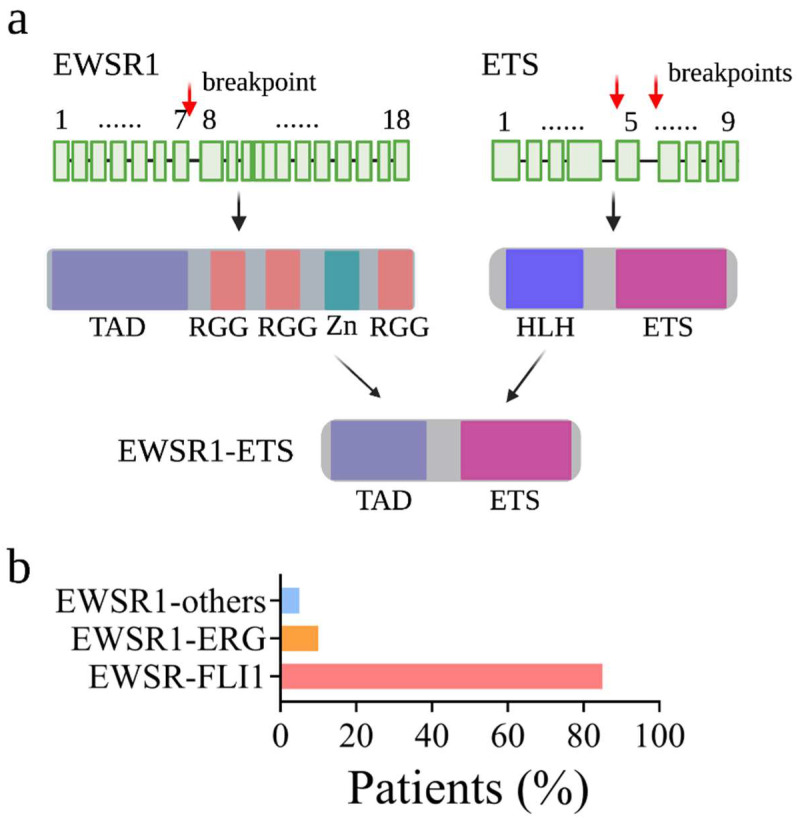
Schematic depiction of fusion protein EWSR1-ETS expressed from fusion genes generated by chromosomal translocation t(11;22)(q24;q12). (**a**) EWSR1 is composed of a transcription activation domain (TAD), three RGG domains, and a Zinc finger domain (Zn). ETS family members have a highly conserved ETS domain and a helix-turn-helix (HLH) structure. The fusion protein is induced by the fusion of the N-terminal TAD of EWSR1 and the C-terminal ETS domain of FLI1. Red arrows indicate the chromatin breakpoints frequently observed in the EwS patients; (**b**) Frequency of EWSR1-ETS fusions identified in EwS patients. Over 85% of EWSR1-ETS fusions involve the FLI1 gene. Around 10% of the EWSR1-ETS fusions are generated by ERG gene.

At the cellular level, EwS has a poorly differentiated stem cell-like phenotype with some degree of neurogenic features [3,4,5]. Histologically, EwS cells appear as uniformly undifferentiated small round cells containing a vesicular nucleus and a small cytoplasm with a sparse intercellular stroma [6]. Morphologically, EwS cells strike a delicate balance between proliferative growth and metastatic capacity along the mesenchymal differentiation axis. Genetically, EwS cancer cells typically exhibit limited numbers of genetic mutations at low frequencies: TP53 (5–10%), CDKN2A (10%), and STAG2 (15%) [7,8]. This is in contrast to tumors in adults, which are primarily caused by accumulated recurrent mutations. However, somatic chromosomal translocations which fuse the Ewing Sarcoma Breakpoint region 1 (EWSR1) gene with a member of the ETS transcription factor family are always observed in EwS. In over 85% of cases, the t(11;22) (q24;q12) translocation fuses the 5′ end of the EWSR1 to the 3′ end of the FLI1 (Friend leukemia integration 1 transcription factor). The translocation gives rise to the EWSR1-FLI1 (also known as EWS-FLI1) fusion protein (Figure 1).

Since EWSR1-FLI1 was identified in the 1990s [9], many studies have shown that the fusion protein EWSR1-FLI1 is often the only genetic alteration detected in EwS and that EWSR1-FLI1 expression is sufficient to lead to the onset and progression of EwS. Inactivation of EWSR1-FLI1 leads to the inhibition of EwS cell growth, the reduction of colony formation on soft agar, and the prevention of tumor formation in immunodeficient mice [10]. Moreover, transformation induced by ectopic expression of EWSR1-FLI1 has also been observed in various types of cells including fibroblasts, mesenchymal stem cells, and myoblastic cells [11,12,13,14,15,16,17,18].

Acting as an aberrant oncogenic transcription factor, EWSR1-FLI1 binds to DNA via the FLI1′s ETS domain and induces gene expression via the EWSR1 amino terminal TAD domain [2]. Mounting evidence also revealed that, in addition to binding to chromatin, EWSR1-FLI1 changes the epigenetic state to alter the gene expression in the cell. Studies aimed at understanding EWSR1-FLI1-mediated cancer susceptibility, prognosis, and gene expression have been conducted at both epigenome and transcriptome levels. For example, ChIP-seq(chromatin immunoprecipitation coupled with high throughput sequencing)-based epigenetic profiling has been conducted to investigate how EWSR1-FLI1 binds to chromatin [19,20] and how EWSR1-FLI1 establishes an appropriate chromatin state for gene expression [21,22]. Using microarrays, several studies defined the transcriptional profiling induced by EWSR1-FLI1 in a variety of cellular models [4,13,17,23,24]. Using RNA-seq(RNA-sequencing)-based transcriptional profiling, other studies compared the gene expression changes between EwS cells with and without EWSR1-FLI1 knockdown [6,25,26]. To fully elucidate gene activation and repression mechanism induced by EWSR1-FLI1 and its chromatin remodeling events, this review discusses the available data with a particular focus on the epigenomic and transcriptomic aspects. Moreover, we review reported high throughput screens conducted in EwS.

## 2. The Epigenetic Regulation Mediated by EWSR1-FLI1

### 2.1. Epigenetic Regulation

In eukaryotes, DNA is packaged into chromatin through interacting with histones [27,28]. Chromatin organization, in turn, is controlled by factors that modify the constituent DNA and histones around which the DNA is packaged [29]. For example, DNA methylation alters chromatin organization by changing the accessibility of DNA [30]. Histones can be post-translationally modified by chromatin modifiers (also known as chromatin modifying factors). Chromatin modifiers can be divided into “writers”, which add modifications to DNA or histones, “erasers”, which remove such modifications, and “readers”, which translate the modifications into cellular responses. Histone modifications change the chromatin compaction level, which alters the binding capacity of transcription factors on chromatin and thus changes the gene expression. In addition to DNA and histone modifications, chromatin 3D structures can also determine the chromatin organization. For example, by forming chromatin loops, the cohesion complex establishes the interaction between enhancers and promoters to maintain chromatin accessibility and gene transcription during cell division [31].

Mounting evidence has shown that dysregulated epigenetic modifications are as significant as genetic mutations and can act as oncogenic driver lesions causing an autonomous growth of cancer cells [32]. A better understanding of epigenetic control of gene expression has begun to provide mechanistic insight into the complex regulatory elements that promote both normal and tumor cell identity and proliferation alike. More importantly, changing of the epigenetic state can alter the expression of multiple oncogenes simultaneously. Therapeutics that target the epigenetic modulators therefore represent powerful alternative treatments for cancer. This is also true for EWSR1-FLI1-induced EwS in which EWSR1-FLI1 reprograms the epigenome to alter the gene expression [2]. Accordingly, understanding the epigenetic regulatory mechanism mediated by EWSR1-FLI1 possesses indispensable significance for EwS treatment.

As described above, EWSR1-FLI1 not only binds to chromatin but also changes the chromatin state to rewire cells for malignancy. Studies have sought to identify EWSR1-FLI1 binding sites on chromatin and map the epigenetic state associated with EWSR1-FLI1. To this end, it has been revealed that EWSR1-FLI1 binds not only to promoters in the vicinity to the transcription start sites (TSSs) but also to enhancers at a long distance [20,22]. At the promoter regions, EWSR1-FLI1 acts as a transcription factor initiating gene expression; while, at the enhancer regions, EWSR1-FLI1 establishes a highly cell-type-specific enhancer to facilitate gene expression [22]. Moreover, at the promoter regions, EWSR1-FLI1 uses a binary-switch model to separate its roles in either activating or repressing gene expression [33]. This decision could be dictated by a specific EWSR1-FLI1 response element present, nearby bound transcriptional regulators, and the local chromatin environment. At some regions, EWSR1-FLI1 binding is associated with open chromatin, nucleosome depletion, reduced DNA methylation, and thus high gene expression, while at other regions, EWSR1-FLI1 binding is associated with condensed chromatin and gene repression.

### 2.2. EWSR1-FLI1 Binding Sites on Chromatin

Since 2008, several research groups have demonstrated that EWSR1-FLI1 uses GGAA-containing elements (microsatellites) as specific response elements to up-regulate genes dispersed throughout the human genome [19,20,34,35]. At least three consecutive GGAA motifs are required for EWSR1-FLI1 binding. Upon binding to GGAA microsatellites, EWSR1-FLI1 increases chromatin accessibility, creates de novo enhancers, and thus transcriptionally activates gene expression [21]. GGAA microsatellites, therefore, act as enhancer elements to provide a high level of transcriptional activity for adjacent target genes. The binding affinity of EWSR1-FLI1 and the gene activation levels positively correlate the numbers of GGAA repeats at the gene promoter regions. A follow-up study found that EWSR1 provides EWSR1-FLI1 fusion protein the capacity to bind to GGAA repeats [36]. Moreover, the chromatin binding capacity provided by EWSR1 only exists when EWSR1 is fused with FLI1. The GGAA-microsatellites binding property is thus a neomorphic property of EWSR1-FLI1. Recently, in the attempt to characterize how EWSR1-FLI1 binds to GGAA microsatellites, two types of microsatellites were identified: “promoter-like” and “enhancer-like” microsatellites [37]. More recent studies also revealed that GGAA microsatellites are essential for EWSR1-FLI1-mediated transcription activation [38]. Indeed, silencing GGAA repeats impaired local nascent transcription and led to a reduced expression of putative target genes. However, it should be noted that the activation of GGAA microsatellites is highly specific to cells expressing EWSR1-FLI1, since these sites remain in a closed chromatin conformation in all other non-EWSR1-FLI1 cells. Therefore, GGAA-microsatellites and their target genes are considered specific targets for EwS treatment.

**Figure 2:**
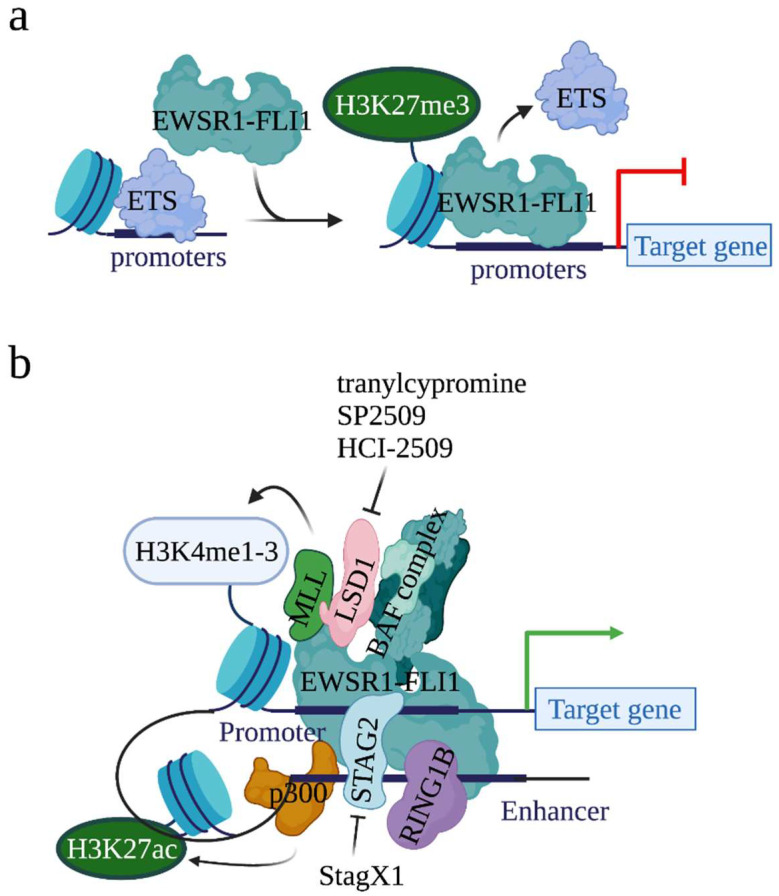
Model of EWSR1-FLI1 in modifying gene expression. (**a**) at the canonical ETS binding sites, in the presence of EWSR1-FLI1, EWSR1-FLI1 displaces ETS transcription factor on the chromatin to repress gene expression; (**b**) STAG2 establishes an enhancer/promoter loop on chromatin. At the enhancer regions, EWSR1-FLI1 interacts with acetyltransferase p300 to establish open chromatin state. RING1B, a catalytic component of the Polycomb-repressive complex 1 (PRC1), also interacts with EWSR1-FLI1 at the enhancers. It should be noted that, when interacting with EWSR1-FLI1, RING1B switches from a transcription repressor to a transcription activator. At the promoter regions, EWSR1-FLI1 recruits histone modifiers MLL, LSD1, BAF complex to establish open chromatin state to initiate gene transcription. Small molecule tranylcypromine, SP2509, and HCI-2509 can be used to target LSD1. Small molecule StagX1 was identified to treat EwS possessing mutant STAG2 by targeting mutant STAG2.

The binding of EWSR1-FLI1 at canonical ETS binding sites (where ETS transcription factors bind) has also been described in both non-malignant endothelial cells and malignant EwS cells [20,21]. At the canonical ETS binding sites, EWSR1-FLI1 represses but not activates the expression of genes (Figure 2a). Functional analysis found that, at the canonical ETS binding sites, EWSR1-FLI1 blocks the expression of genes that are either tumor suppressors or mesenchymal lineage regulators in non-EwS contexts. Moreover, the binding of ETS transcription factors on chromatin was dramatically increased when EWSR1-FLI1 was depleted, suggesting that EWSR1-FLI1 displaces the wild type ETS transcription factor and thus inactivates the conserved enhancers to repress gene expression. Together, EWSR1-FLI1 induces gene expression when it binds to the GGAA microsatellites, whereas EWSR1-FLI1 represses gene expression when it binds to the canonical ETS binding sites.

### 2.3. EWSR1-FLI1 Establishes Open Chromatin State at GGAA Microsatellites

To map chromatin context that are EWSR1-FLI1, Riggi et al., compared the EWSR1-FLI1 binding sites with four histone modifications H3K4me1, H3K4me2, H3K4me3, and H3K27ac using ChIP-seq [21]. They found that the majority of EWSR1-FLI1 binding sites display H3K4me1, a ubiquitous marker of cis-regulatory elements, and H3K27ac, a more specific marker of enhancer activation. In addition, all promoters bound by EWSR1-FLI1 harbor H3K4me3, a marker of transcriptional initiation. These observations indicate that EWSR1-FLI1 binds cis-regulatory elements to regulate gene expression.

To investigate the underlying regulatory mechanism of how EWSR1-FLI1 alters epigenetic states, Tomazou et al., mapped the epigenome in a cellular model that is dependent on EWSR1-FLI1 and compared the epigenome states of EWSR1-FLI1-high and EWSR1-FLI1-low EwS cells [22]. In this study, the authors analyzed the DNA methylation using bisulfite sequencing, checked the histone modifications (H3K4me3, H3K4me1, H3K27ac, H3K56ac, H3K36me, H3K27me3, and H3K9me3) using ChIP-seq, measured the open chromatin status using ATAC-seq, and identified the transcription changes using RNA-seq. They found that EWSR1-FLI1 binding is associated with reduced DNA methylation, opened chromatin, depleted nucleosome, and elevated gene expression. These observations highlight that EWSR1-FLI1 establishes active promoter/enhancer signatures to initiate transcription at genes that would otherwise be silent in these cells.

### 2.4. EWSR1-FLI1 Alters Epigenetic State by Recruiting Chromatin Modifiers to Its Binding Sites on Chromatin

EWSR1-FLI1 can alter chromatin states, although it does not have histone modification activity. For example, the expression of EWSR1-FLI1 in primary pediatric mesenchymal stem cells (MSCs) increased H3K4me1 and H3K27ac modification levels at EWSR1-FLI1 binding sites. On the contrary, the knockdown of EWSR1-FLI1 in EwS cells reduced H3K27ac levels at EWSR1-FLI1 binding sites. To understand how EWSR1-FLI1 alters histone modifications, several studies have demonstrated that EWSR1-FLI1 acts as a pioneer factor that recruits histone methyltransferases and acetyltransferases on chromatin to modify histones (Figure 2b).

#### 2.4.1. Histone Acetyltransferase p300

In 2014, Riggi et al., showed that 75% of activated EWSR1-FLI1 binding sites were enriched with acetyltransferase p300, a chromatin regulator which acetylates H3K27 [21] (Figure 2b). Further, when EWSR1-FLI1 was inactivated, due to the loss of p300 on chromatin, H3K27ac was not detected. These results suggest that EWSR1-FLI1 recruits p300 to acetylate H3K27 at its binding sites. Additionally, the same study observed the binding of the MLL (mixed-lineage leukemia) complexes, a complex family which methylates H3K4, at EWSR1-FLI1 binding regions. Together, EWSR1-FLI1 recruits p300 and the MLL complexes at its binding loci to acetylate H3K27 and methylate H3K4, respectively, generating an open chromatin status to initiate gene transcription.

#### 2.4.2. BAF (BRG1/BRM-Associated Factor) Chromatin Remodeling Complex

Also known as SWI/SNF (switch/sucrose non-fermentable) complex, BAF is another complex which can be recruited by EWSR1-FLI1 onto chromatin [39] (Figure 2b). The BAF complex regulates gene expression by altering genomic architecture and DNA accessibility [40]. Oncogenic programs driven by mutated BAF complex have been observed in 20–25% of all human cancers. In an attempt to identify proteins interacting with the BAF complex, EWSR1 was identified [39]. Direct interaction between the BAF complex and EWSR1-FLI1 was detected by Co-immunoprecipitation (Co-IP). Overlap between BAF binding sites and EWSR1-FLI1 binding sites on chromatin was observed at the genome wide level in EwS cells. Further, the knockdown of EWSR1-FLI1 depleted the binding of the BAF complex at GGAA microsatellites, leading to the conclusion that EWSR1-FLI1 recruits the BAF complex to chromatin. More importantly, inactivation of BAF reduced the expression of EWSR1-FLI1 target genes, suggesting that EWSR1-FLI1 utilizes the BAF complex to activate genes and establish a tumor-specific transcriptional program.

#### 2.4.3. LSD1 (Lysine-Specific Demethylase 1)

LSD1 (also known as KDM1A) is another histone modifier that interacts with EWSR1-FLI1 [41] (Figure 2b). As the first identified histone demethylase, LSD1 demethylates H3K4me1/2, H3K9me1/2, H4K20me1/2, and other non-histone targets, such as p53 and DNMT1 [42]. By modifying the chromatin status, LSD1 controls broad expression programs. In cancers, a high expression of LSD1 has been observed in a variety of cancers, including EwS, and the high expression correlates with lower overall survival [43,44,45,46]. In EwS, throughout the genome, EWSR1-FLI1 and LSD1 co-localize at both EWSR1-FLI1-binding GGAA microsatellites and non-microsatellites which span in the promoter, intronic, and intergenic regions [43,47]. Further investigation showed that EWSR1-FLI1 drives dynamic genome-wide reorganization of LSD1 and recruits LSD1 to mediate epigenetic and transcriptional changes [48]. Due to its role in modifying the chromatin state at the EWSR1-FLI1 binding sites and EWSR1-FLI1-mediated gene expression, LSD1 has thus been considered a valuable therapeutic target for EwS treatment [49]. Several studies have shown that treatment with LSD1 inhibitors, e.g., tranylcypromine, SP2509, and HCI-2509, reversed the EWSR1-FLI1 transcription signature and impaired the growth of EwS cell lines [50,51].

#### 2.4.4. RING1B (Ring Finger Protein 2)

Another well-studied EWSR1-FLI1 interacting chromatin modifier is RING1B [52,53]. Acting as the catalytic component of the Polycomb-repressive complex 1 (PRC1), RING1B represses gene expression by catalyzing H2A K119 ubiquitination [54]. However, it has also been shown that RING1B transcriptionally activates gene expression when it interacts with UTX, an H3K27 demethylase, and acetyltransferase p300 [55]. High expression of RING1B in EwS tumors in an EWSR1-FLI1-independent manner was reported [52]. Direct interaction between EWSR1-FLI1 and RING1B was revealed by Co-IP. ChIP-seq analysis observed the binding of RING1B at both active enhancers of actively transcribed genes and repressive promoters of transcriptionally repressed genes [53]. At regions where RING1B binds, and where EWSR1-FLI1 does not, RING1B represses gene transcription. However, at regions where both RING1B and EWSR1-FLI1 bind, RING1B acts as a recruiter of EWSR1-FLI1 to chromatin, thus facilitating EWSR1-FLI1-mediated gene activation (Figure 2b).

#### 2.4.5. STAG2 (Stromal Antigen 2)

As described above, EWSR1-FLI1 not only binds to promoters but also enhancers. STAG2 has been identified as critical for the binding of EWSR1-FLI1 at the super enhancer regions and EWSR1-FLI1-mediated transcription [56,57]. STAG2 is a member of the cohesin complex that forms a ring structure around the enhancer and promoter regions [58]. The cohesin complex holds sister chromatids together during mitosis and, more importantly, regulates gene expression through the integrated promoter/enhancer complex [59]. In cancers, STAG2 is frequently mutated [60,61]. In EwS, STAG2 mutation (15–20%) is the second most frequent genetic alteration, following EWSR1-FLI1 (~85%) [2,7]. Mechanism analysis found that STAG2 mutation disrupts the formation of CTCF/cohesin chromatin loops at the promoter–enhancer regions [57]. Together, at enhancers, STAG2 governs and establishes a gene-regulatory architecture to modulate EWSR1-FLI1-mediated oncogenic and developmental programs. In line with this idea, Zhang et al., conducted a high throughput small molecule compound screen to identify compounds that exhibit lethality in the context of EwS cells expressing mutant STAG2 [56,62]. Consequently, they identified an isoquinolinone compound, StagX1, which exhibits specificity in inhibiting proliferation of STAG2-mutated EwS cells but not EwS cells without STAG2 mutation (Figure 2b and Table 1).

#### 2.4.6. HDACs (Histone Deacetylases)

By removing acetylation from proteins including histones, HDACs increase chromatin condensation to repress gene expression. It has been observed that EWSR1-FLI1 could recruit HDACs to gene promoter regions to conduct its gene repression function [33,63]. Consequently, HDAC inhibitors reactivate the expression of EWSR1-FLI1 suppressed genes and inhibit EwS growth [64]. For example, entinostat and sodium butyrate, two HDAC inhibitors, suppress EwS tumor growth by inducing the expression of p21 and TGFBR2, two EWSR1-FLI1-repressed genes [65,66,67]. Further mechanistic studies also revealed that sodium butyrate could lead to EwS death by inhibiting EWSR1-FLI1-mediated differentiation suppression in a general manner [68].

## 3. The Transcriptional Regulation Mediated by EWSR1-FLI1

As introduced above, the expression of EWSR1-FLI1 in EwS results in a potent chimeric oncoprotein with novel biological properties and a unique transcriptional signature essential for oncogenesis. Mechanistically, EWSR1-FLI1 could act as both a transcription activator and a transcription repressor; while both functions are essential for EWSR1-FLI1 in mediating oncogenesis. By altering gene expression at the molecular level, EWSR1-FLI1 plays roles in a variety of cellular processes including cell cycle, apoptosis, metabolism, angiogenesis, chromosome segregation, and cell migration. Identification of dependencies incurred by EWSR1-FLI1 expression would offer a compelling avenue for the development of effective targeted therapies. Previous reports have described that rather than inducing specific signaling pathways, EWSR1-FLI1 activates a larger set of genes which are involved in a variety of cellular processes. Here, we summarize the studies that characterized genes whose expressions are directly regulated by EWSR1-FLI1.

### 3.1. EWSR1-FLI1 Regulates Gene Transcription by Interacting with Non-Chromatin Modifers on Chromatin

#### 3.1.1. E2F Family Transcription Factors

**Figure 3:**
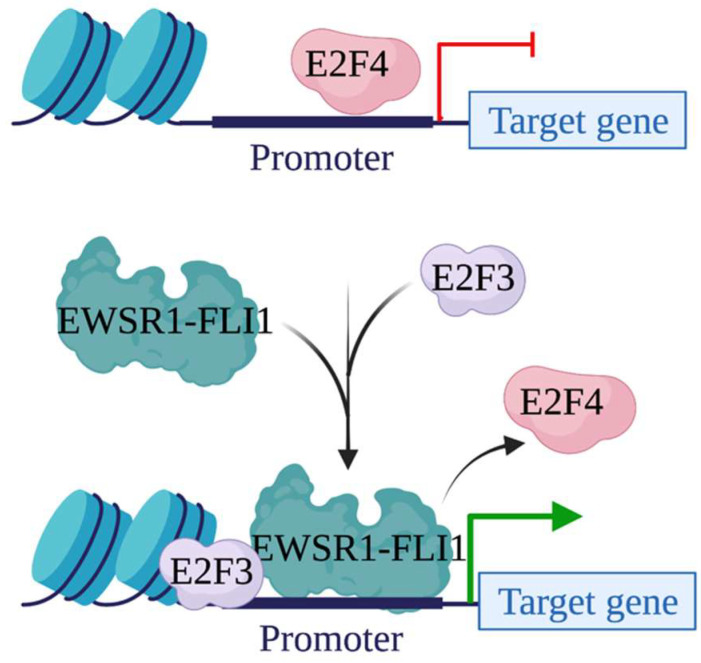
EWSR1-FLI1 employs an E2F switch to drive target gene expression. In the absence of EWSR1-FLI1, E2F4 binds to chromatin to repress gene expression. In the presence of EWSR1-FLI1, E2F3 displaces E2F4 and recruits EWSR1-FLI1 on chromatin to initiate gene transcription.

Studies have found that EWSR1-FLI1 can regulate gene transcription by interacting with other transcription effectors on chromatin. For example, it has been reported that EWSR1-FLI1 uses transcription factors E2Fs to facilitate its target gene transcription [69]. There are eight E2F genes in humans: E2F1-3 are transcription activators, while E2F4-8 are transcription repressors [70]. E2Fs regulate a variety of cellular responses and thus play a pivotal role during cancer development [71]. Dr. Heinrich Kovar’s group in Austria characterized the interplay between EWSR1-FLI1 and E2Fs. They found that EWSR1-FLI1 up-regulated genes are enriched with E2Fs regulated genes due to the co-localization of EWSR1-FLI1 and E2F3 at TSSs regions [72]. More detailed analysis found that the binding of E2F3 at EWSR1-FLI1 occupancy loci exists at the TSSs of genes which are up-regulated by EWSR1-FLI1 but not those of genes which are down-regulated by EWSR1-FLI1. Functional analysis found that, after being transcriptionally induced by EWSR1-FLI1, E2F3 cooperates with EWSR1-FLI1 to facilitate EWSR1-FLI1-mediated gene activation. More interestingly, the binding of E2F4 was also observed at EWSR1-FLI1 and E2F3 co-localization sites [73]. Further analysis revealed that EWSR1-FLI1 employs an E2F switch to drive target gene expression. In the presence of EWSR1-FLI1, E2F3 takes over the binding of E2F4 on chromatin and interacts with EWSR1-FLI1 to initiate target gene transcription (Figure 3). In the absence of EWSR1-FLI1, E2F4 replaces E2F3 to restrain the binding of EWSR1-FLI1 on chromatin and thus inhibit gene transcription.

#### 3.1.2. NR0B1 (Nuclear Receptor Subfamily 0, Group B, Member 1)

Another protein which helps EWSR1-FLI1-mediated transcription is NR0B1 (also known as DAX1) [19,74,75,76]. Acting as an orphan nuclear hormone receptor, NR0B1 plays roles in steroid hormone production, osteoblast differentiation, sex determination, and maintenance of stem cell property [77]. High expression of NR0B1 was observed in EwS but not non-EwS cancer cells [75]. Revealed by Co-IP and ChIP-seq assays, NR0B1 physically interacts with EWSR1-FLI1 on chromatin to promote cell-cycle progression and EWSR1-FLI1-mediated oncogenic transformation [19,76,78]. Moreover, NR0B1 is also a direct target of EWSR1-FLI1 [79]. Taken together, NR0B1 facilitates EWSR1-FLI1-mediated tumorigenesis by promoting cell-cycle progression using a feed forward regulatory mechanism.

#### 3.1.3. RHA (RNA Helicase A)

EWSR1-FLI1 also directly interacts with RHA, a member of the DEXH box helicase family of proteins, to regulate transcription and RNA splicing [80]. RHA, also known as nuclear DNA helicase II or DHX9, is an integral component of protein complexes that regulate transcription and splicing. RHA was initially identified as an EWSR1-FLI1 binding partner from a phage library screening [80]. Further study found that the interaction between EWSR1-FLI1 and RHA is in an RNA-dependent manner [81]. Functional analysis found that, when it binds to EWSR1-FLI1, RHA acts as transcriptional co-activator to augment EWSR1-FLI1 in activating gene expression. A knockdown of RHA led to a decrease of EwS cell viability. In line with this discovery, EWSR1-FLI1-mediated tumor growth can be impaired when the interaction between EWSR1-FLI1 and RHA was disrupted by the pharmacologic inhibitor YK-4-279 [82]. This study provided proof of concept evidence that blocking the interaction of mutant cancer-specific transcription factors with their binding partners could provide a promising strategy for developing the tumor type-specific anticancer agents [83]. More recently, a Phase I/II trial in relapsed/refractory EwS patients reported that TK-216, a clinical derivative of YK-4-279, can be combined with other chemotherapeutic agents such as vincristine for future EwS treatment [84].

### 3.2. Roles of EWSR1-FLI1 in Activiating Gene Expression

#### 3.2.1. NR0B1

As described in 3.1.2., while functioning as one of the EWSR1-FLI1-interacting proteins, NR0B1 was identified as one of the highest up-regulated EWSR1-FLI1 targets [19,74,75,79]. The induction of NR0B1 by EWSR1-FLI1 was demonstrated by two independent research groups using two different experiment designs. As observed by Mendiola et al., the ectopic expression of EWSR1-FLI1 induced the NR0B1 expression in several cell models [75]. Kinsey et al., showed that the inactivation of EWSR1-FLI1 reduced the expression of NR0B1 at both mRNA and protein levels in EwS cells [74]. Moreover, the binding of EWSR1-FLI1 was detected at the NR0B1 promoter region, demonstrating a direct regulation of NR0B1 expression by EWSR1-FLI1 [78]. Finally, functional analysis found that NR0B1 is required for EWSR1-FLI1-mediated oncogenic transformation as the inactivation of NR0B1 impaired EwS cell growth in vitro and tumorigenesis in immunodeficient mice.

#### 3.2.2. EZH2 (Enhancer of Zeste)

Acting as a direct downstream target of EWSR1-FLI1, EZH2 maintains the stemness feature of EwS cells [11,12,85,86]. EZH2 is a component of the Polycomb repressor complex 2 (PRC2) and it suppresses gene expression by methylating lysine 27 on histone 3 (H3K27) [87]. The roles of EZH2 have been implicated in stem cell maintenance, embryonic development, and cell differentiation [88]. In cancers, an abnormally increased EZH2 expression is correlated with poor prognosis [89,90,91]. In EwS, EWSR1-FLI1 binds to the EZH2 promoter and induces expression of EZH2 at the transcriptional level [86]. Gene expression profiling performed in primary EwS tumor and in stem cells expressing EWSR1-FLI1 found that, downstream of EZH2, HOX genes disrupt developmental transcription programs and thus provide the stemness feature for EwS tumors [12]. Inactivation of EZH2 in EwS cells impaired contact-independent growth in vitro and tumorigenic growth in vivo, suggesting that EZH2 can serve as a therapeutic target. Mechanistically, the inhibition of EZH2 increased the expression of genes involved in neuroectodermal and endothelial differentiation (EMP1, EPHB2, GFAP, and GAP43) [85]. In sum, downstream of EWSR1-FLI1, EZH2 prevents EwS cell differentiation and maintains a stem cell-like state, resulting in oncogenic transformation and tumor progression.

#### 3.2.3. NKX2.2 (NK2 Homeobox 2)

Demonstrated by Stephen Lessnick’s group, NKX2.2 is another gene that is highly up-regulated by EWSR1-FLI1 [26,63,92]. Acting as a homeodomain-containing transcription factor, NKX2.2 is involved in many developmental contexts [93]. In 2006, NKX2.2 was identified as an EWS/FLI-regulated gene that is necessary for oncogenic transformation in EwS [26]. In this study, endogenous EWSR1-FLI1 was knocked down by RNAi and then exogenous EWSR1-FLI1 was expressed. From this, NKX2.2 was identified as an EWSR1-FLI1 up-regulated gene. Functional analysis revealed that the knockdown of NKX2.2 impaired the oncogenic transformation in soft agar assays and impaired the tumor development in the xenograft model of EwS. Follow-up studies found that NKX2.2-repressed genes are enriched in the EWSR1-FLI1-repressed dataset that contributes to mesenchymal differentiation and terminal differentiation [63,92]. More recently, studies found that NKX2.2 binds to the promoter region of STEAP1 (six transmembrane epithelial antigen of the prostate 1), a gene which is essential for EwS survival [94], together with EWSR1-FLI1 to regulate expression of STEAP1 [95]. Although this study did not highlight the direct interaction between NKX2.2 and EWSR1-FLI1 on the chromatin in the genome-wide level, it provided some understanding of how NKX2.2 coordinates the effects of EWSR1-FLI1. Due to its unique expression in Ewing tumors, several studies have shown that NKX2.2 is a valuable immunohistochemical marker for EwS detection, with higher sensitivity and specificity than other EWSR1-FLI1-induced genes, including NR0B1, E2F3, and EZH2 [96,97,98,99,100].

#### 3.2.4. KDM3A (Lysine Demethylase 3A)

KDM3A (also known as JMJD1A) has also been identified as one of the critical mediators for EWSR1-FLI1-drived EwS growth [101,102,103]. KDM3A, one of the Jumonji-domain histone demethylases (JHDMs), removes methyl groups from mono- and di-methylated H3K9 [104]. EWSR1-FLI1 induces the expression of KDM3A by releasing the suppression of miRNA-22 on KDM3A [101]. Upon activation, KDM3A up-regulates oncogenes, including Cyclin D1 (CCND1) and insulin growth factor 1 receptor (IGF1R), which have each been identified as up-regulated oncogenes in EwS, to generate the tumorigenesis propensity. Further studies found that the knockdown of KDM3A in EwS cells decreased the cell migration in vitro and decreased metastasis in vivo by downregulating genes involved in metastasis [103]. More recently, the inhibition of KDM3A using its pharmacologic inhibitor JIB-04 has shown tumor suppression effects [102,105]. KDM3A, therefore, represents a valuable target for EwS treatment.

#### 3.2.5. GLI1 (Glioma-Associated Oncogene Homolog 1)

Another critical factor for EWSR1-FLI1-mediated tumor development is GLI1 [106,107,108]. GLI1 is the principal transcriptional effector of the canonical Hedgehog (HH) pathway [109]. Acting as a transcription factor, the roles of GLI1 have been implicated in stem-cell maintenance and developmental processes. The HH-GLI1 pathway has been correlated to tumorigenesis and aggressive phenotypes of several cancer types [110]. The transcriptional up-regulation of GLI1 induced by EWSR1-FLI1 was initially observed in NIH 3T3 cells expressing EWSR1-FLI1. The inactivation of GLI1 impaired anchorage-independent growth of NIH 3T3 cells expressing EWSR1-FLI1. The induction of GLI1 by EWSR1-FLI1 in multiple EwS cellular models and primary tumor specimens was also reported by several research groups [106]. The binding of EWSR1-FLI1 at the GLI1 promoter region was observed and the inactivation of EWSR1-FLI1 abolished GLI1 expression, indicating that GLI1 is a direct EWSR1-FLI1 target [107]. Importantly, several genes known to be transcriptionally modulated by EWSR1-FLI1 are dependent upon GLI1 expression, suggesting that it is the central role of GLI1 for EWSR1-FLI1-mediated tumorigenesis. Functional analysis revealed that the knockdown of GLI1 is sufficient to impair the capacity of EwS cells for anchorage-independent growth and the colony formation in EwS cells [108]. The pharmacological inhibition of GLI1 using the compound GANT58 and the small molecule NCS75503 resembles the GLI1 knockdown-induced growth defect in EwS cells [107]. Most importantly, arsenic trioxide (ATO), an anticancer drug that inhibits cell growth by targeting GLI1 [111,112], significantly inhibited the metastasis capability of EwS cells in vitro and reduced the tumor burden in 75% of stage III EwS patients [113].

#### 3.2.6. Homeobox Protein MEIS1

Due to the binding of EWSR1-FLI1 at super enhancers, EWSR1-FLI1 target genes are enriched with super-enhancer transcripts. For example, MEIS1 has been identified as one of the super-enhancer transcripts that is transcriptionally induced by EWSR1-FLI1 in EwS [114]. MEIS1 is a homeodomain transcription factor belonging to the Three Amino Acid Loop Extension (TALE) family of homeodomain-containing protein. Acting as a co-factor of homeobox (HOX) family members, MEIS1 has been extensively studied for its essential role in hematopoietic cells differentiation [115]. Mechanism analysis found that MEIS1 co-localizes with EWSR1-FLI1 on chromatin to co-operate EWSR1-FLI1-mediated transcription [114]. The knockdown of MEIS1 led to cell apoptosis in vitro and inhibited xenograft tumor growth in vivo. On the contrary, over-expression of MEIS1 enhanced colony growth of EwS cells. Together, in EwS, enhancers are associated with EWSR1-FLI1 binding, enhanced gene expression, and re-enforced EwS cancer cell state, and super-enhancer transcripts can be leveraged to identify novel oncogenes in EwS.

#### 3.2.7. IL1RAP (Interleukin 1 Receptor Accessory Protein)

To identify regulators which are essential for metastasis, Zhang et al., utilized proteomic approaches and revealed a significant up-regulation of IL1RAP in cells resistant to anoikis (detachment-induced death) [116]. From this study, IL1RAP was identified as a direct target of EWSR1-FLI1 and a key driver of metastasis in EwS. Best known as a co-receptor for IL1R signaling, IL1RAP mediates signaling triggered by interleukin-1 (IL1) [117]. In vivo, ILR1AP depletion blocked primary tumor growth at primary implantation sites and impaired the tumor invasiveness into neighboring organs. Mechanistic analysis found that IL1RAP enhances cysteine uptake and glutathione antioxidant by interacting with CD98 and the cysteine transporter. Therapeutically, inhibiting IL1RAP induced EwS cell death by disrupting metabolic homeostasis in the cells. This study uncovered that IL1RAP could be used as a cell-surface therapeutic target to block EwS progression.

In addition, it has also been documented that EWSR1-FLI1 activates platelet-derived growth factor (PDGF) [118] and cMYC to stimulate cell proliferation [119], up-regulates ID2 to evade growth inhibition [119], and increases VEGF expression to induce angiogenesis [120]. By doing so, EWSR1-FLI1 has high propensity for tumor development and progression.

### 3.3. Roles of EWSR1-FLI1 in Repressing Gene Expression

#### 3.3.1. TGFBR2 (Transforming Growth Factor Beta Receptor 2)

The first gene that was identified to be suppressed by EWSR1-FLI1 is TGFBR2 [121], which encodes the TGF-β type II receptor. EWSR1-FLI1 binds the promoter region of TGFBR2 to directly repress TGFBR2 expression [33]. TGFBR2 functions as a tumor suppressor. Consequently, ectopic expression of TGFBR2 suppressed the in vivo growth of EwS cells. In sum, the suppression of TGFBR2 mediated by EWSR1-FLI1 is essential for EWSR1-FLI1-mediated transformation.

#### 3.3.2. IGFBP3 (Insulin Like Growth Factor Binding Protein 3)

In 2004, Prieur et al., found that IGFBP3 was up-regulated when EWSR1-FLI1 was inactivated by siRNA, suggesting that the expression of IGFBP3 is suppressed by EWSR1-FI1 in EwS cells [25]. The direct binding of EWSR1-FLI1 at the IGFBP3 promoter region was observed from a luciferase assay. Further, it has also been shown that expression of EWSR1-FLI1 in HeLa cells, a non-EwS context, also inhibited expression of IGFBP3. Therefore, EWSR1-FLI1 suppresses IGFBP3 in a general manner.

#### 3.3.3. LOX (Lysyl Oxidase)

By comparing EWSR1-FLI1 transcriptional profile data and genome-wide EWSR1-FLI1 binding sites on chromatin, Stephan Lessnick’s group found that there is a large set of genes that are repressed by EWSR1-FLI1 and characterized the transcriptional repressive function of EWSR1-FLI1 [33]. For example, Sankar et al., showed that EWSR1-FLI1 binds to the LOX promoter region directly to suppress its expression. LOX, encodes Lysyl oxidase, acts as a tumor suppressor in several cancers, including EwS [122]. A low expression of LOX was observed in EwS and the ectopic expression of LOX impaired xenograft tumor formation in vivo [33]. Mechanism characterization showed that EWSR1-FLI1 recruits the transcriptional repressor complex NuRD to the LOX promoter region, leading to histones deacetylation and thus expression repression.

#### 3.3.4. FOXO1 (Forkhead Box O1)

The enrichment of forkhead box (FOX) recognition motifs was observed in the EWSR1-FLI1-repressed gene set [123]. Consequently, EWSR1-FLI1-mediated down-regulation of FOXO1 in EwS was reported. As a transcription factor, FOXO1 regulates cell processes such as glucose hemostasis, cell cycle, and apoptosis by regulating gene expression directly [124]. Additional studies in EwS found that EWSR1-FLI1 binds at the promoter region of FOXO1 [125], suggesting that the repression of FOXO1 in EwS is directly regulated by EWSR1-FLI1. Functional analysis found the ectopic expression of FOXO1 induced EwS cell death in vitro and decreased EwS tumor growth in the xenograft mouse model.

Other EWSR1-FLI1-repressed genes also include the tumor suppressor SPRY1 [126] and p21 [67]. Acting as a downstream target of fibroblast growth factor receptor (FGFR), SPRY1 inhibits MAPK by inhibiting the phosphorylation of ERK to prevent tumor growth [127]. In EwS patients, SPRY1 expression positively correlates with improved survival, suggesting a tumor suppression role in EwS [126]. The induction of SPRY1 in EwS cells impaired proliferation, clonogenic growth, and migration. Although binding of EWSR1-FLI1 at the SPRY1 promoter region was not detected, up-regulation of SPRY1 was observed in EwS cells in which EWSR1-FLI1 was knocked down, suggesting that SPRY1 is an EWSR1-FLI1 regulated target. P21 is a cyclin-dependent kinase inhibitor that suppresses the G1-S cell cycle transition [128]. In EwS, although functions of p21 in regulating EwS development have not been characterized, it has been revealed that EWSR1-FLI1 binds to the promoter region of p21 to negatively regulate its expression [67].

## 4. High Throughput Screens in EwS

Studies using either gain-of-function or loss-of-function strategies are essential to decipher roles of the specific epigenetic and transcriptional alterations for cancer growth and survival. In recent years, high throughput biochemical and genetic screens enable us to systematically identify functional mechanisms and novel vulnerabilities in cancers in an unbiased manner. Here, we summarize the high throughput chemical screens, RNA interference (RNAi) screens, and CRISPR-Cas9 screens conducted in EwS (Table 1).

**Table 1:** High throughput functional screens conducted in EwS.

Types	Aims	Inhibitors and Targets	Libraries	References
Chemical screens	Identify compounds to attenuate EWSR1-FLI1 activity	Cytosine arabinoside (ARA-C)	1040 cpds (NCI)	Stegmaier et al., 2007 [129]
		Mithramycin	50,000 cpds (NCI)	Grohar et al., 2011 [130]
		Midostaurin	1280 cpds (Sigma)	Boro et al., 2012 [131]
	Identify compounds to alter activity FAK, a highly activated kinase in EwS	AURKB inhibitor AZD-1152	1912 cpds (MIPE 4.0)	Wang et al., 2019 [132]
	Identify compounds to inhibit growth of mutant STAG2 EwS cells	StagX1	8000 cpds	Zhang et al., 2022 [62]
RNAi screens	Identify kinase targets involved in EwS growth	STK10 and TNK2	Kinase siRNA library (572 genes)	Arora et al., 2010 [133]
	Identify regulators which modulate EWSR1-FLI1 activity	HNRNPH1 and SF3B1	Genome-scale siRNA library	Grohar et al., 2016 [134]
	Identify regulators involved in EWSR1-FLI1 driven cell viability	LRWD1	Druggable siRNA library (6781 genes)	He et al., 2016 [135]
CRISPR-Cas9 screens	Identify genetic dependencies specific for growth of TP53 wild-type EwS cells	MDM2, MDM4, USP7, and PPM1D	Genome-scale CRISPR-Cas9 library	Stolte et al., 2018 [136]
	Identify regulators which alter EWSR1-FLI1 stability	TRIM8		Seong et al., 2021 [137]
	Identify genes whose knockout conferred resistance to the LSD1 inhibition in EwS	Mitochondrial electron transport chain complexes III and IV		Tokarsky et al., 2022 [138]

### 4.1. Chemical Screens Conducted in EwS

In 2007, Stegmaier et al., performed a small molecular library screen to identify modulators of EWSR1-FLI1 activity in EwS [129]. In this study, 1040 small molecules, including many FDA-approved drugs, from the National Institute of Neurological Disorders and Stroke bioactive small molecule library were screened. This study found that, cytosine arabinoside (ARA-C; also known as cytarabine), a drug already used to treat children with leukemia, might also be effective in the treatment of EwS. Treatment with ARA-C abrogates EwS cells anchorage-independent growth and reduced xenograft tumor growth in vivo. Mechanism analysis found that ARA-C attenuates EWSR1-FLI1 transcription signature by decreasing the EWSR1-FLI1 expression level. In a study by Grohar et al., over 50,000 compounds were tested for their ability to inhibit EWSR1-FLI1 activity in TC32 EwS cells [130]. As a result, mithramycin, a drug which binds to GC-rich regions of the genome and inhibits the SP1 family of transcription factors, was identified. Treatment of mithramycin impaired the growth of EwS xenograft tumors but not the growth of osteosarcoma xenograft tumors, suggesting that mithramycin may be used to selectively treat EwS.

As an extension of the Stegmaier et al., study, Boro et al., screened a small molecule compound library enriched for FDA-approved drugs to identify the one with the capacity to modulate the expression of EWSR1-FLI1 target genes [131]. This study identified a kinase inhibitor, midostaurin, and found that midostaurin treatment induced apoptosis and thus suppressed tumor growth in the EwS xenograft model. Midostaurin is currently undergoing phase II clinical trials for treatment of leukemias and the results of this study support the initiation of clinical trials of midostaurin in children with EwS.

Recently, Wang et al., conducted a high throughput screen of 1912 compounds in combination with the FAK inhibitor, PF-562271, to identify new combination treatment approaches with FAK inhibition [132]. From this study, Aurora kinase inhibitors were identified to induce the synergistic inhibition of EwS cell viability. In particular, Aurora kinase B (AURKB) inhibitor AZD-1152 sensitizes EwS cells to treatment using PF-562271. In vivo, the combination of FAK and AURKB inhibition improved the survival of mice harboring the EwS xenograft tumor.

More recently, Zhang et al., screened 8000 compounds to identify inhibitors for the treatment of EwS cells harboring mutant STAG2 [62]. From this study, an isoquinolinone compound, StagX1, was identified as a selective inhibitor that inhibits the growth of EwS cells expressing mutant STAG2 but not wild type STAG2. The detailed mechanisms of how StagX1 inhibits the growth of mutant STAG2 EwS cells have not been well-characterized. However, the high selectivity of StagX1 in blocking the growth of mutant STAG2 EwS cells but not the growth of all other types of cancer cells may provide StagX1 an advantage for use as a specific therapeutic agent.

### 4.2. RNA Interference-Based Screens Conducted in EwS

In 2010, Arora et al., identified essential kinases that regulate the growth and survival of EwS cells using an siRNA library targeting 572 human kinases [133]. To this end, along with MK-STYX and AKT1, two reported EWSR1-FLI1 targets, STK10 and TNK2 were identified to be important in the growth of EwS cells. STK10, a serine/threonine kinase highly expressed in lymphocytes, plays an important role in cell growth and morphogenesis. TNK2, a specific CDC42 partner, has been shown to be involved in cell migration and induction of metastasis in transformed cells.

In 2016, Grohar et al., conducted a genome-wide RNAi screen to identify therapeutic vulnerabilities in EWSR1-FLI1 activity [134]. They found that genes associated with mRNA splicing and processing are important for EWSR1-FLI1 activity. The knockdown of SF3B1 (splicing factor 3b subunit 1) or HNRNPH1 (heterogeneous nuclear ribonucleoprotein H1), two components of spliceosome, led to selective changes in the expression of genes that are deregulated in EwS by disrupting splicing of the EWSR1-FLI1 transcript. Mechanism characterization found that HNRNPH1 mediates the splicing of EWSR1-FLI1, whereas SF3B1 is required for the generation of an EWSR1-FLI1 transcript that includes all exons. This study provides a potential strategy for the treatment of EwS through disruption of the processing of the EWSR1-FLI1 transcript.

In 2017, He et al., performed a siRNA-based high throughput screen (6781 human genes were targeted, 4 siRNA molecules per target gene) to identify genes whose function is critical for EWSR1-FLI1 driven cell viability [135]. This study found that the knockdown of LRWD1 (leucine rich repeats and WD repeat domain containing 1) impaired EwS cell viability in an EWSR1-FLI1 dependent manner. Mechanism characterization revealed that LRWD1 acts as an EWSR1-FLI1 direct downstream target to repress differentiation and thus maintain the stemness state of EwS cells.

### 4.3. CRISPR-Cas9-Based Screens Conducted in EwS

In 2018, Stolte et al., used a genome-scale CRISPR-Cas9 screen to discover targets specific for TP53 wild type EwS [136]. They found that MDM2, MDM4, USP7, and PPM1D can be used as therapeutic targets for TP53 wild type EwS in a selective manner. Inhibition of any of these four genes using either a genetic or a chemical approach impaired viability of TP53 wild type but not TP53 mutated EwS cells. Moreover, the inhibition of MDM2 and MDM4 with ATSP-7041, a MDM2/4 dual inhibitor, synergized with the inhibition of USP7. Most importantly, chemically inhibiting these proteins in combination provided the most effective mechanism to trigger TP53-mediated cell death in EwS. This study highlighted the potential of genetic screens to predict synergistic drug combination and developed combinational therapies for TP53 wild type EwS.

In 2021, Seong et al., performed a genome-scale CRISPR-Cas9 screen to identify regulators which control EWSR1-FLI1 protein stability [137]. From this study, they found that the knockout of tripartite motif-containing 8 (TRIM8) increases EWSR1-FLI1 at the protein level but not at the mRNA level. A high expression of EWSR1-FLI1 leads to EwS cell death by exacerbating DNA damage. Consequently, the knockout of TRIM8 led to reduced tumor growth in the xenograft model. Mechanism analysis found that, acting as an E3 ligase which ubiquitinates target protein for degradation, TRIM8 mediates the degradation of EWSR1-FLI1, indicating that EwS possesses a selective dependency on TRIM8. This study highlighted the fusion oncoprotein-specific degradation pathways as selective therapeutic targets.

More recently, another genome-scale CRISPR-cas9 screen was conducted to identify genes whose knockout conferred resistance to the LSD1 inhibitor SP-2509 with the aim of developing effective combination strategies [138]. This study found that the depletion of genes in mitochondrial electron transport chain complexes III and IV confer resistance to SP-2509, suggesting that mitochondrial dysfunction mediates SP-2509 drug resistance. Currently, an analog of SP-2509, known as seclidemstat, is in clinical trials to treat relapsed or refractory EwS. Together, this study suggests that the combination of LSD1 inhibitors with agents which prevent mitochondrial dysfunction would provide more efficacy for EwS treatment.

## 5. Discussion and Perspectives

The development and implementation of genome-wide approaches enables us to characterize the epigenetic and transcriptional regulation mediated by EWSR1-FLI1 in a comprehensive manner. To this end, it has been revealed that EWSR1-FLI1 binds to both GGAA microsatellites and the canonical ETS binding sites on chromatin [19,20]. Upon binding to chromatin, EWSR1-FLI1 interacts and thus recruits other proteins, such as histone modifiers [21,39], transcription factors [69], and RNA helicase A [80], to modulate its downstream gene expression. It should be noted that GGAA-mediated gene transcription is specific to EwS. Therefore, genes transcriptionally induced by activated GGAA-microsatellites represent a tumor-specific mechanism that may be exploited for the development of targeted therapies for EwS treatment. Moreover, it has also been observed that EWSR1-FLI1 interacting proteins can be used as specific therapies for EwS treatment.

An improved understanding of the molecular biology of EwS is essential for developing novel therapies. Considerable work has gone into the identification of the transcriptional targets of EWSR1-FLI1. A larger set of genes have been identified as important for EWSR1-FLI1-mediated tumorigenesis. However, despite the effort, very limited targets have been clinically demonstrated to be of prognostic or therapeutic significance. It has been shown that EwS cells are of significant heterogeneity and EwS cells switch between functionally distinct cell states dependent on EWSR1-FLI1 fluctuations. Cells with high EWSR1-FLI1 expression levels proliferate exponentially, whereas cells with low EWSR1-FLI1 expression tend to be migratory and invasive [139]. The heterogeneity and tumor cell plasticity enables EwS cells to adapt to the environment via transcriptomic and metabolic alternations [140,141]. Therefore, there is still an urgent need for further functional studies to fully understand the dynamics and the clinical impacts of EwS development and progression. These studies could help to identify new therapeutics that target EWSR1-FLI1-mediated epigenetic and transcriptional signaling with better efficacies either alone or in combination with standard of care chemotherapeutic treatments.

Since 2007, several studies have conducted high throughput chemical, RNAi, and CRISPR-Cas9 screens to identify therapeutic targets in an unbiased manner [62,129,130,131,132,133,134,135,136,137,138,142,143]. These screens are also being used to determine genes involved in drug resistance and to identify synergistic drug regimens that are more potent than equally effective doses of its components. Successful combination therapies may also aid in more manageable side effects through reduced dosing, providing additional benefits for treatment of tumors in children, who are less tolerant of adverse side effects. High throughput chemical and genetic screens thus represent one of the emerging fields in EwS research that deserves additional attention.

Taken together, studies on EWSR1-FLI1-induced transcriptional and epigenetic changes have shed light on the initiation and progression of EwS. Furthermore, the implementation of high through chemical/genetic screens highlighted exciting opportunities for future EwS treatments. We also foresee the adaptation of state-of-the-art technologies such as the CRISPR gene tiling scan and single-cell CRISPR screens [144] in the EwS field to discover more in-depth mechanistic and translational insights into the disease etiology and therapeutic opportunities in EwS.

## Data Availability

Not applicable.
